# Cell death as an architect of adult skin stem cell niches

**DOI:** 10.1038/s41418-024-01297-3

**Published:** 2024-04-22

**Authors:** Kim Lecomte, Annagiada Toniolo, Esther Hoste

**Affiliations:** 1 VIB Center for Inflammation Research, 9052 Ghent, Belgium; 2 Department of Biomedical Molecular Biology, Ghent University, 9052 Ghent, Belgium

**Keywords:** Cell biology, Cell death and immune response

## Abstract

Our skin provides a physical and immunological barrier against dehydration and environmental insults ranging from microbial attacks, toxins and UV irradiation to wounding. Proper functioning of the skin barrier largely depends on the interplay between keratinocytes- the epithelial cells of the skin- and immune cells. Two spatially distinct populations of keratinocyte stem cells (SCs) maintain the epidermal barrier function and the hair follicle. These SCs are inherently long-lived, but cell death can occur within their niches and impacts their functionality. The default cell death programme in skin is apoptosis, an orderly and non-inflammatory suicide programme. However, recent findings are shedding light on the significance of various modes of regulated necrotic cell death, which are lytic and can provoke inflammation within the local skin environment. While the presence of dying cells was generally regarded as a mere consequence of inflammation, findings in various human dermatological conditions and experimental mouse models of aberrant cell death control demonstrated that cell death programmes in keratinocytes (KCs) can drive skin inflammation and even tumour initiation. When cells die, they need to be removed by phagocytosis and KCs can function as non-professional phagocytes of apoptotic cells with important implications for their SC capacities. It is becoming apparent that in conditions of heightened SC activity, distinct cell death modalities differentially impact the different skin SC populations in their local niches. Here, we describe how regulated cell death modalities functionally affect epidermal SC niches along with their relevance to injury repair, inflammatory skin disorders and cancer.

## Facts

- Cell death is a central element in healthy mammalian skin and regulates hair cycling
- Distinct types of regulated cell death differentially alter the various stem cell niches present in skin
- Dysregulated cell death pathways in keratinocytes drive skin inflammation and carcinogenesis
- Keratinocyte stem cells can act as occasional phagocytes by clearing dead/dying cells

## Outstanding Questions

- Which factors define the differential sensitivity of distinct skin stem cells to cell death?
- Is there a role for non-apoptotic cell death programmes in hair cycling regulation?
- Can we manipulate cell death responses to improve cutaneous wound healing, while preventing tumorigenesis?
- What is the therapeutic potential of manipulating cell death switches in the skin?

## The epidermis as a structural and immunological barrier

Skin comprises an epidermal layer, consisting of keratinocytes (KCs) and various immune cell-types including innate immune cells such as Langerhans cells and macrophages, but also adaptive memory T-cells and cells considered to bridge innate and adaptive immunity, namely γδ T-cells. Underneath the epidermal layer, the skin’s dermis is mainly populated by fibroblasts, adipocytes and endothelial cells [1]. KCs form the structure of the epidermis and its appendages, including hair follicles (HFs), sweat glands and sebaceous glands [2]. KCs located in the interfollicular epidermis maintain the permeability barrier of the skin, crucial to sustain the hydration levels of the body. This barrier is formed by differentiating KCs originating from the keratinocyte stem and progenitor cells, which are located in the basal layer -the lower layer of the epidermis where cells are attached to the basement membrane. Asymmetric SC divisions in this basal layer lead to the detachment of one of the daughter cells from this membrane, which results in the gradual differentiation of KCs to form a cornified layer protecting the skin from excessive water loss, UVB irradiation and pathogen entry [3]. This terminal differentiation process is also termed ‘cornification’ and is tightly orchestrated as reviewed elsewhere [4]. KCs, dermal fibroblasts and resident immune cells can release various cytokines, chemokines, danger-associated molecular patterns (DAMPs) and antimicrobial peptides to evoke an appropriate immune response upon skin challenge [1]. These molecules will bind their cognate receptors leading to the production of more inflammatory mediators and cytoprotective factors, thereby fighting off infections, while assisting in the maintenance of the skin barrier function. This intercellular crosstalk between KCs, fibroblasts and immune cells is crucial to mediate skin function, not only in homeostasis, but also in injury repair and other inflammatory conditions [1, 5].

## Distinct skin stem cell niches exhibit different sensitivities to cell death

**Fig. 1:**
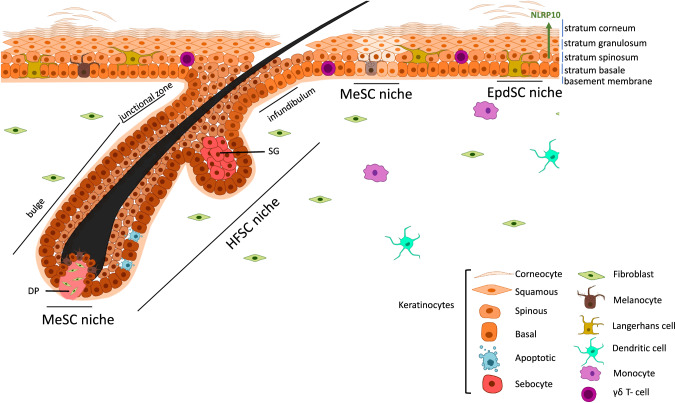
Different SC populations in adult mouse skin and the major cell death programmes described in their niches. The interfollicular epidermis of the skin consists of KCs, maintained by EpdSCs, and various immune cells. These SCs are attached to the basement membrane and give rise to transient amplifying cells capable to differentiate into corneocytes. In this epidermal niche, cell death is actively suppressed to ensure proper epidermal barrier formation, although recent findings describe the existence of specific NLRP10 inflammasome complexes, mediating pyroptotic cell death, in the differentiating epidermal layers [46]. HFs present in the skin are maintained by a specific population of SCs (HFSCs). In the HFSC niche, a substantial amount of KCs undergo periodic bouts of proliferation and subsequent apoptotic cell death. These apoptotic cells are phagocytosed by neighbouring basal KCs [23], ensuring absence of a significant immune response in this natural cycling process. Melanocyte stem cells (MeSCs) also populate the skin, and apoptosis of these cells has been linked to pigmentation disorders, such as vitiligo. SG sebaceous gland, DP dermal papilla.

As a tissue with a remarkably high epithelial turnover rate, skin depends on a delicate balance between cell proliferation, differentiation, senescence and death. To provide for the specific proliferative needs of each epidermal compartment, mammalian skin contains different SC populations that are spatially separated in distinct niches [2]. These niches are not merely physical locations harbouring SCs, but refer to the microenvironment where intrinsic and extrinsic signals, including those emanating from dying/dead cells, converge to determine SC fates. Keratinocyte SCs residing in the basal layer of the interfollicular epidermis, termed epidermal stem cells (EpdSCs), proliferate to give rise to KCs that can differentiate into corneocytes and thereby maintain the barrier function of the skin [2]. Cell death is normally absent in the various layers of homeostatic interfollicular epidermis [6], indicating robust pro-survival mechanisms that facilitate the entry of KCs into the differentiation process and ensure successful completion of barrier formation (Fig. 1). Indeed, genetic mouse models have elucidated that aberrant cell death in the EpdSC niche often results in barrier perturbation and subsequent inflammation [7–10] (Table 1). A second population of keratinocyte SCs is located in the hair follicle (HF). These hair follicle stem cells (HFSCs) can be discriminated in mouse skin based on specific marker gene expression, such as *Lgr5*, *Cd34* and *Sox9* [11]. HFSCs also depend on their attachment to the basement membrane to maintain stemness, and undergo a specialized differentiation programme to ensure HF maintenance during the various stages of the hair cycle [12–14]. The spatially distinct niche where HFSCs reside is known as the bulge (Fig. 1). In contrast to the EpdSC niche, this tissue compartment experiences periodic bouts of substantial cell death during hair cycle regression (Box 1). In between EpdSCs and HFSCs, a region known as the junctional zone, LRIG1+ keratinocyte SCs are present and give rise to the murine sebaceous gland. A subpopulation of these SCs expresses GATA6, a marker for the sebaceous duct [15, 16]. The distinct populations of keratinocyte SCs are spatially and functionally separated in homeostatic skin conditions. However, during injury repair and in neoplastic conditions, several HFSCs are mobilized to regions where they normally do not reside to exert functions that differ from their homeostatic roles, a phenomenon that is termed SC plasticity [17–19].

**Table 1:** Summary of keratinocyte-selective knock-out mice with skin phenotypes steered by aberrant cell death control.

KC-specific KO	Skin phenotype	Targeted signalling pathway affecting cell death control	Refs
*A20 (Tnfaip3)*	Dishevelled hair, sebocyte hyperplasia	TNF-mediated signalling	[72]
*Arts (Sept4)*	Elevated number of HFSCs, accelerated wound healing	Apoptosis	[137]
*Atg7*	Thickening of cornified layer	LC-3 dependent autophagy	[126, 127]
*Atg9a*	Dermatitis, enhanced KC apoptosis	LC-3 independent autophagy	[128]
*Atg16L1*	Thickening of cornified layer, more KC apoptosis, precocious hair cycling, accelerated wound healing	LC-3 dependent autophagy	[25]
*Bax in HFSCs (on Bak−/− backgound)*	Increased HFSC compartment, accelerated wound healing	Intrinsic apoptosis	[60]
*Casp-8*	Dermatitis, early postnatal lethality, KC necroptosis	Apoptosis, necroptosis, pyroptosis	[9, 10]
*Casp-9 in HFSCs*	Accelerated wound repair, de novo HF regeneration	Intrinsic apoptosis	[139]
*cFLIP*	Dermatitis, early postnatal lethality, KC apoptosis	Apoptosis, necroptosis	[66, 67]
*FADD*	Dermatitis, early postnatal lethality, KC necroptosis	Extrinsic apoptosis	[8]
*Gpx4*	Focal alopecia, dysmorphic hair, dermatitis	Ferroptosis	[32]
*Hmgb1*	Accelerated wound healing, protection from tumour formation	NET formation in neutrophils	[132]
*Hoil-1*	Dermatitis, early postnatal lethality	Apoptosis, necroptosis	[79]
*Hoip*	Dermatitis, early postnatal lethality	Apoptosis, necroptosis	[79]
*Ikk1*	Impaired epidermal barrier function, early postnatal lethality	TNF-mediated signalling	[71]
*Ikk2*	Dermatitis, early postnatal lethality	TNF-mediated signalling	[65]
*Nemo*	Dermatitis, enhanced TNF-dependent KC apoptosis, loss of pigmentation, early postnatal lethality	TNF-mediated signalling	[70]
*Nlrp10*	Enhanced sensitivity to contact hypersensivity	Pyroptosis	[46]
*Otulin*	Dermatitis, spontaneous tumour formation	Apoptosis, necroptosis	[78, 87]
*Rel-a and c-Rel*	Dermatitis	TNF-mediated signalling	[68]
*Ripk1*	Dermatitis, KC necroptosis and apoptosis	apoptosis, necroptosis	[7]
*Sharpin*	Dermatitis, KC apoptosis	Apoptosis	[86]
*Tak1*	Dermatitis, enhanced KC cell death	TNF-mediated signalling	[69]
*Traf2*	Psoriasis-like dermatitis, enhanced KC cell death	TNF-mediated signalling	[73]

Hair cycling is characterized by a growth phase of the HF (anagen), followed by a regression phase (catagen) mediated by excessive keratinocyte death, and a subsequent resting phase (telogen) [20] (Box 1). Growth of the HF is induced by reciprocal interactions between HFSCs and fibroblasts located in the dermal papilla, a specialized structure at the base of the HF [2, 21]. After growth, the length of the HF drastically reduces in the late stages of catagen, a process mediated by cell death and contraction of the dermal sheath, composed of smooth muscle cells wrapping the HF [20, 22]. Despite the extensive cell death that occurs in catagen, this physiological process is not accompanied by a pronounced inflammatory response. This implicates apoptosis, as a non-lytic cell death modality, as a major cell death programme in catagen [20]. Indeed, elegant intravital imaging studies demonstrated a spatial gradient of apoptotic basal KCs during the regression stage of the HF [23]. Signalling downstream of tumour necrosis factor-receptor-1 (TNFR1), a crucial decision point between pro-inflammatory signalling or cell death induction, has been implicated in hair cycling [24–26]. Not only is the death of KCs essential for regulating hair cycling, but apoptotic macrophages expressing TREM2 also influence HFSC proliferation (Box 1) [27]. Clearly, the distinct SC niches present in skin are confronted with varying rates of cell death, to which they respond differentially in order to maintain their specific tissue compartments.

Other SC populations present in the skin of most mammals include melanocyte stem cells (MeSCs) responsible for skin pigmentation, and sweat gland SCs [2, 28]. While limited information exists on the role of different cell death programmes in melanocyte and sweat gland biology, the death of melanocytes is associated with the common pigmentation disorder vitiligo. Moreover, genome-wide association studies (GWAS) have identified variations in genes involved in the executioner phase of apoptosis as susceptibility factors for vitiligo [29]. Here, we focus on examining the impact of cell death on keratinocyte SC niches in homeostasis and during injury repair and tumour formation, as recent advances highlight the crucial role played by various cell death programmes and their control mechanisms in preserving skin function in these conditions.

Box 1: Schematic representation of the different stages of the hair cycle

In mammalian skin, the coordinated death of keratinocytes is a central element in the regulation of HF regression. HFs cycle throughout life and can be in a state of active proliferation, termed anagen. During this stage, the HF protrudes deep into the dermis and facilitates hair growth. Induction of anagen depends on cell death responses in macrophages and keratinocytes present in the HFSC niche [23, 27]. Upon completion of anagen, a portion of keratinocyte SCs located in the HF die simultaneously in a cyclic manner. The regression stage of the HF is termed catagen and is facilitated by TNF-dependent apoptosis [24–26]. During catagen a large portion of HF cells are killed. However, a limited population of outer root sheet cells remain intact and migrate to the bulge to restrict HFSC activation [158]. Indeed, following regression, the HF enters a resting stage termed telogen during which HFSCs are quiescent.

## Regulated cell death in the skin

**Fig. 2:**
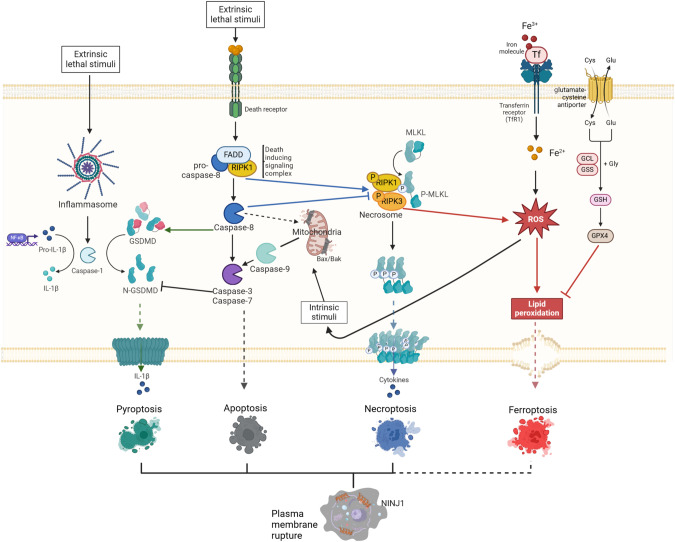
Overview of the most prominent cell death modalities described in keratinocytes. Apoptosis is the primary cell death modality in homeostatic keratinocytes. Intrinsic apoptosis is initiated by activation of the BCL-2 family members BAK and BAX, mediating mitochondrial leakage resulting in cytochrome c release into the cytosol. This event triggers formation of the apoptosome which assembles and activates caspase-9. Subsequently the executioner caspases-3 and -7 are cleaved and activated, mediating cell demise. Extrinsic apoptosis is triggered by binding of toxic agents to death receptors. This results in the assembly of a complex containing adaptor proteins (such as FADD and TRADD) and caspase-8, followed by cleavage of caspase-8 and subsequently of caspase-3 and -7. In conditions where apoptosis is blocked, binding of death receptors can lead to induction of necroptosis. This is mediated by RIPK3 phosphorylation leading to phosphorylation of MLKL, pore formation and cell lysis. Keratinocytes can die by pyroptosis through DAMP or PAMP recognition. Pyroptosis is preceeded by inflammasome formation. In this multi-protein complex, caspase-1 is assembled and cleaved, an event that leads to cleavage of pro-IL-1β and pro-IL-18 into their mature forms and cleavage of GSDMs, inducing pore formation in the plasma membrane and release of cytokines. Ferroptosis is a regulated cell death programme dependent on iron and resulting in lipid peroxidation of the phospholipids present in the plasma membrane by reactive oxygen species (ROS). GPX4 plays a crucial role in protecting cells from ferroptosis and this enzyme depends on cysteine as a co-factor.

Regulated cell death was long considered synonymous to apoptosis, but different types of molecularly controlled cell death pathways, including necroptosis, pyroptosis and ferroptosis, have now been described and implicated in the maintenance of skin immune homeostasis [7, 30–32]. Apoptosis is mediated by upstream initiator caspases (caspase-8 and -9), which activate downstream effector caspases (caspase-3, -6 and -7) responsible for executing the apoptotic process. This process is typically non-immunogenic due to the formation of apoptotic bodies that are phagocytosed by neighbouring cells [23]. Indeed, efficient removal of these cell corpses, a process termed efferocytosis, will prohibit the induction of a pronounced immune response [33]. Conversely, necroptosis and pyroptosis are lytic types of cell death that generate pores in the plasma membrane leading to the release of the intracellular content, including cytokines and DAMPs, in the extracellular environment. This induces a strong inflammatory response (Fig. 2) [34, 35]. Lytic cell death can potently drive skin inflammation, but upon certain challenges KCs can opt to undergo senescence, a cellular programme that is also capable of instigating inflammatory responses (Reviewed in [36]). The relative contribution of these two biological processes to dermatitis remains to be determined, but will likely depend on the nature of the inflammatory trigger.

Necroptosis is induced in conditions of deregulated apoptotic signalling, upon triggering of death receptors, type 1 interferon receptor, specific Toll-like receptors or infections with pathogens [37–41]. This type of programmed death relies on the activation of receptor interacting protein kinase 3 (RIPK3) and subsequent phosphorylation of mixed-lineage kinase domain-like protein (MLKL), resulting in MLKL translocation to the plasma membrane, subsequent pore formation and cell lysis [42]. Dysregulated apoptotic signalling can lead to sensitization of KCs to necroptosis and proper control of necroptosis in this cell-type is crucial to prevent dermatitis [7, 8]. Pyroptosis is another form of lytic cell death, usually caused by microbial infection, that relies on the proteolytic activation of gasdermin-D (GSDMD) by caspase-1 and caspase-11 or caspase-8, thereby unleashing GSDMD’s pore-forming activity [43]. Next to MLKL and GSDMD facilitating pore formation in different cell death types, plasma membrane rupture is further accomplished by NINJURIN-1 [44]. Activation of caspase-1 occurs in inflammasome complexes that are assembled after triggering of specific NOD-like receptors (NLRs), AIM2 (absent in melanoma 2) or pyrin. These receptors recognize various microbial insults or other stressors including extracellular ATP and uric acid crystals [45]. Cells dying by pyroptosis release bioactive IL-1β and IL-18, two cytokines that potently alter skin immunity. Increasing evidence suggests that inflammasome complexes play a crucial role in mediating skin immune surveillance [29, 30, 46]. Other forms of cell death have been described more recently in the context of cutaneous pathology, including ferroptosis, NETosis, autophagy-induced cell death, cuproptosis and panoptosis [47–49]. These cell death programmes will only be briefly discussed here, as their roles in skin homeostasis and inflammation are largely enigmatic.

### Apoptosis and necroptosis

Apoptosis was the first type of cell death shown to be regulated by specific genetic programmes [50]. This cell death modality regulates important developmental processes and is crucial for tissue homeostasis [51, 52]. Although apoptotic cells can release compounds that affect tissue functioning [53], apoptosis is generally considered immune silent. However, an excessive amount of apoptosis can lead to secondary necrosis and subsequent inflammation [54, 55]. In homeostatic skin conditions, apoptotic caspases are not activated in the EpdSC niche [6]. Conversely, a marked abundance of apoptosis is induced in the HFSC niche during catagen, the regression stage of the hair cycle (Box 1). These cyclic bouts of cell death eliminate a substantial number of KCs and melanocytes in the HFSC niche, without provoking significant inflammation. This phenomenon thus offers a unique model for studying how tissue physiology is maintained in the presence of abundant cell death.

**Fig. 3:**
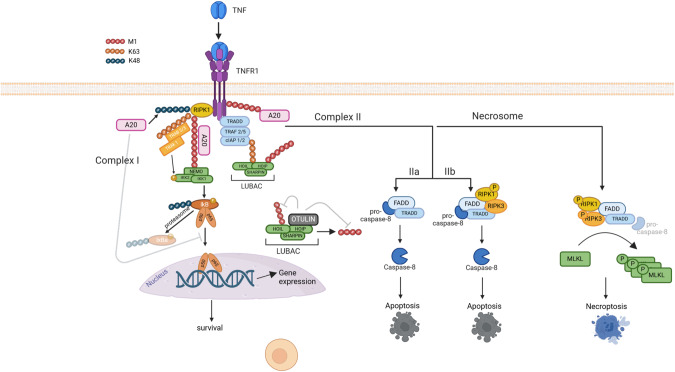
Schematic overview of TNFR-mediated signalling. Binding of TNF to TNFR1 results in the downstream formation of multi-protein complexes that mediate either survival, apoptosis or necroptotic cell death. Formation of complex I at the plasma membrane results in activation of NF-kB signalling and transcription of pro-inflammatory and pro-survival genes. In conditions where complex I is disassembled, a subsequent complex II is formed, resulting in apoptosis. When caspase-8 is absent or inhibited, the necrosome complex is assembled, leading to phosphorylation of RIPK3, followed by phosphorylation of MLKL and pore formation in the plasma membrane.

Apoptosis is induced by two different pathways, depending on the origin of the trigger (Fig. 2). The intrinsic pathway to apoptosis occurs upon dysregulation of intracellular homeostasis caused by toxic triggers or DNA damage, and involves BAX/BAK-induced mitochondrial pore formation and cytochrome c release from the mitochondria [56]. This induces the formation of the apoptosome and activation of caspase-9 [57]. In conditions of DNA damage triggering intrinsic apoptosis, the longevity of HFSCs is regulated by overexpression of the anti-apoptotic protein BCL-2 [58]. Upregulation of this protein in mouse KCs leads to abnormal HFSC niche architecture, evidenced by the enlarged bulge area that develops over time in these mice [59]. In agreement, depletion of the pro-apoptotic protein BAX in HFs results in HFSC niche expansion [60]. The extrinsic apoptotic pathway is triggered by cell death stimuli of the TNF superfamily, including TNF, Fas ligand (FASL) and TNF-related apoptosis-inducing ligand (TRAIL) that bind to their cognate receptors on the plasma membrane. Binding of TNF to TNFR1 results in the induction of a pro-inflammatory signalling cascade leading to nuclear factor-kappa B (NF-κB) activation and survival, or in the formation of a death-inducing complex. The decision between these paradoxical outcomes depends on the assembly of specific multi-protein complexes [61] (Fig. 3). Formation of complex I involves the recruitment of TRADD and RIPK1 to TNFR1, allowing the subsequent assembly of TRAF2/5, cIAP1/2 and the Linear Ubiquitination Chain Assembly Complex (LUBAC), activating a downstream signalling cascade leading to NF-κB and MAPK activation, induction of pro-inflammatory signalling and survival. The ubiquitin network associated with complex I is negatively regulated by A20, CYLD and OTULIN, which destabilize the signalling complex and attenuate signalling to MAPKs and NF-κB [61]. In a pro-survival context, caspase-8 can cleave RIPK1 thereby limiting apoptotic and necroptotic cell death [62]. However, TNF can also induce cell death via the assembly of a secondary cytosolic complex, termed complex II, which originates from the binding of FAS-associated death domain-containing protein (FADD) to the receptor-dissociated complex I components TRADD and/or RIPK1. This complex now functions as a platform for binding and activation of caspase-8 triggering apoptotic or pyroptotic cell death, depending on the cellular context [63, 64]. In the absence of caspase-8 activation, a different complex termed the necrosome can be assembled, involving RIPK1 and RIPK3, which drives necroptosis. The significance of precisely controlling the formation of TNF-induced complexes in KCs is evident from the severe inflammatory skin phenotypes that arise after depletion of the proteins constituting these complexes or the checkpoints regulating their activity in mouse KCs [65–73] (Table 1). Additionally, several human skin pathologies are associated with variations in genes responsible for assembling TNF-induced complexes [74].

Genetic ablation of key apoptotic mediators, such as caspase-8, FADD and RIPK1, in mouse KCs triggers KC necroptosis and severe skin inflammation [7–10] (Table 1). The induction of necroptosis in KCs initiates a feed forward cycle of inflammation, subsequent epidermal barrier permeability and skin demise, explaining the perinatal or early postnatal lethality associated with these genotypes. Mice lacking caspase-8 selectively in KCs exhibit hyperplasia of the basal and spinous layers, expression of keratin-6 (K6) in the interfollicular epidermis, enhanced cell death rates in the epidermis and marked infiltration of specific subsets of immune cells in both epidermis and dermis [9, 10]. The epidermis of skin with FADD-deficient KCs shows similar defects [8]. Likewise, mice lacking RIPK1 in KCs exhibit epidermal thickening, reduced K10 expression and abundant presence of apoptotic and necroptotic cells in their epidermis. Ablation of RIPK3 in these KC-selective RIPK1-deficient mice averts skin inflammation, indicating the high potency of necroptosis in driving dermatitis [7]. RIPK1 prevents KC necroptosis in a kinase-independent manner by binding to RIPK3 via RIP homotypic interaction motif (RHIM) domains. Absence of the RIPK1 RHIM domain in KCs leads to spontaneous Z-DNA-binding protein 1 (ZBP1)-dependent necroptosis, severe skin inflammation and perinatal lethality. Indeed, RIPK1 RHIM is essential to prevent ZBP1 from binding and activating RIPK3 and subsequent MLKL phosporylation [75–77]. Interestingly, when mice with these highly inflamed skin phenotypes due to absence of apoptotic genes are born, inflammation seems to be increased from postnatal day 5 onwards, coinciding with the initiation of the first round of anagen [8, 78, 79]. It is therefore tempting to speculate that the altered activation state of the HFSCs contributes to the inflammatory phenotypes observed in these mice.

Whether KCs die by apoptosis or necroptosis has pivotal implications for skin function. Whitin the TNFR-pathway, various key decision points that switch the cell death programme from apoptosis to necroptosis have been identified [61] (Fig. 3). One of these control mechanisms is ubiquitination, a post-translational modification in which chains of ubiquitin (Ubq) molecules are attached on substrates. This process is mediated by ubiquitin E3 ligases and reversed by deubiquitinases (DUBs) [80]. These Ubq chains are linked by lysine residues present in Ubq or by its N-terminal methionine residue (therefore also termed M1 chains) and have important scaffolding functions for the assembly of the pro-inflammatory complex 1 upon TNF triggering [81]. Linear Ubq chains are attached to their substrates by the E3 ligase complex LUBAC consisting of HOIL-1 (heme-oxidized IRP2 ubiquitin ligase 1), HOIP (HOIL-1 interacting protein) and SHARPIN (Shank-associated RH-domain interacting protein), and are removed by the DUBs CYLD (cylindromatosis) and OTULIN (OTU deubiquitinase with linear linkage specificity) [82–84]. While CYLD can remove both K63 and M1 Ubq chains, OTULIN has exclusive affinity for M1 Ubq chains [83, 85]. Interestingly, genetic deletion of single LUBAC components or of the DUBs that can remove M1 chains, selectively in KCs, results in severe inflammatory skin phenotypes caused by excessive KC death [78, 79, 86, 87] (Table 1). While deletion of HOIP and HOIL-1 in KCs results in massive skin inflammation and early postnatal lethality, which is delayed upon compound removal of TNF or TNFR1 [79], the phenotypes of mice lacking functional SHARPIN or keratinocyte-selective OTULIN are respectively milder or fully rescued upon removal of TNFR1 [78, 84, 87, 88], indicating that different molecular mechanisms regulate KC lethality in these distinct genetic contexts. Importantly, patients carrying loss-of-function mutations in the *OTULIN* gene suffer from ORAS (OTULIN-related autoinflammatory syndrome), a severe and often lethal autoinflammatory syndrome affecting multiple organs including skin. ORAS patients present with skin lesions reminiscent of nodular panniculitis with neutrophil infiltration and apoptotic cells are evident in these lesions [82, 89]. They are successfully treated with anti-TNF therapy [82], further emphasizing that proper control of TNFR-mediated signalling is critical for skin immunosurveillance. It is worth noting that keratinocyte SC niches are highly sensitive to TNF-mediated cell death in OTULIN-deficient conditions, causing autoinflammation and even carcinogenesis due to enhanced apoptotic and necroptotic responses in KCs [78, 87]. This is not the case in other high-turnover SC niches, such as the intestinal epithelium, where OTULIN deficiency does not cause spontaneous pathology [90].

Presence of apoptotic and necroptotic cells has been observed in patients suffering from a range of inflammatory skin diseases, including psoriasis, atopic dermatitis, vitiligo and epidermolysis bullosa [31, 91, 92]. In addition, genetic linkage studies have implicated apoptotic and necroptotic signalling pathways in various dermatological conditions. GWAS identified genetic variations in *TNFAIP3* (encoding A20), *TNIP1* and *FASL*, involved in apoptotic and necroptotic cell death regulation, as susceptibility factors for psoriasis vulgaris [93, 94]. In alopecia areata, a common hair loss disorder associated with autoinflammation, polymorphisms in the anti-apoptotic gene *BCL2L2* have been identified [95]. Most importantly, TNF-neutralizing antibodies are highly successful for clinical treatment of moderate to severe psoriasis [96]. Skin samples from psoriatic patients show elevated levels of several necroptosis-associated proteins, such as RIPK1, RIPK3 and MLKL, and recent findings demonstrated that inhibiting KC necroptosis significantly delays the development of imiquimod-induced psoriasis in mice [91, 97]. Due to the fact that cell death and inflammation are so tightly linked, with cell death being a potential instigator or consequence of inflammation and vice versa, additional studies are needed to reveal which cell death mediators play a causative role in dermatitis. The first oral, selective RIPK1 inhibitor GSK2982772 is currently being tested for treatment of active plaque psoriasis patients, leading to reduced epidermal thickness and lower inflammatory infiltrates [98].

### Pyroptosis

Pyroptosis is an inflammatory lytic cell death programme, characterized by rupture of the cell membrane due to gasdermin-mediated pore formation. Originally described as candidate genes causing hair loss [99], the mouse GSDM superfamily includes *Gsdma* (comprising 3 homologues), -*c* (comprising 4 homologues), -*d* and -*e*, while the human genome encodes for GSDM-A to -E and pejvakin. These proteins require cleavage to translocate and insert into the cell membrane [100–102]. While pyroptosis was initially believed to be a cell death programme exclusive to innate immunity cells like monocytes and macrophages, a surge in genetic evidence now suggests a crucial role for pyroptosis in KC and melanocyte biology [45]. Loss-of-function mutations in the human *NLRP1* (Nod-like receptor family pyrin domain containing 1) gene, a pyroptotic receptor, are the causative factor for vitiligo-associated multiple autoimmune disease. These patients typically suffer from generalized vitiligo often associated with psoriasis or systemic lupus erythematosus [30]. Moreover, gain-of-function (GOF) mutations in *NLRP1* have been identified in patients with dermatological conditions as a result of spontaneous inflammasome activation in KCs [103–105]. The importance of proper control of NLRP1 activity in skin is also apparent in patients carrying hypomorphic or knockout alleles of the NLRP1 inhibitor *DPP9* (dipeptidyl peptidase 9), who present with skin pigmentation abnormalities [106]. Patients suffering from Cryopyrin-associated periodic syndrome (CAPS), due to GOF mutations in the inflammasome receptor *NLRP3*, present with severe cutaneous rashes [107]. Knockin of one such *Nlrp3* mutation in mice phenocopies human CAPS, including severe dermatitis [108, 109]. It is important to stipulate that dysregulated inflammasome signalling can drive altered secretion of IL-1 and IL-18 by various cell-types and is not necessarily linked to enhanced cell death levels in KC compartments. However, mice carrying GOF mutations in mouse *Nlrp3* are protected from dermatitis when crossed onto a GSDMD-deficient background, implicating a pivotal role for GSDMD-mediated pyroptosis in the skin [110]. Also, GSDME-mediated pyroptosis has been shown to protect keratinocytes from UV-radiation and viral infections [111, 112]. A third inflammasome receptor for which genetic variations have been linked to dermatological conditions is PYRIN (encoded by *MEFV*). These mutations recapitulate an immune response triggered by pathogens in skin and patients present with neutrophilic dermatosis, which can be ameliorated by blocking IL-1 signalling [113].

Whole-genome studies in psoriatic patients revealed SNPs (Single Nucleotide Polymorphisms) in *AIM2*, an activator of pyroptosis [114]. Intriguingly, *AIM2* is crucial in establishing inflammatory memory in mouse EpdSCs, enabling them to respond more efficiently to subsequent insults [115]. KCs also express high levels of NLRP10 in the EpdSC niche upon differentiation, driving inflammasome formation in conditions of mitochondrial damage [46]. The role of this inflammasome in mediating cutaneous immunity is also apparent from the enhanced sensitivity of mice with keratinocyte-selective deletion of *Nlrp10* to contact dermatitis [116]. Drugs targeting inflammasome activation or outcome, such as colchicine and Anakinra, are used to treat several inflammatory skin diseases, including neutrophilic dermatoses and hidradenitis suppurativa [117, 118]. Various clinical trials targeting IL-1 family cytokines to ameliorate dermatological conditions have been initiated and are reviewed elsewhere [119].

### Ferroptosis

Ferroptosis is a cell death pathway that critically depends on iron radicals and peroxidation of plasma membrane lipids [120, 121]. Cells undergoing ferroptosis display shrinkage of their mitochondria with an increase in their membrane density and cristae reduction, but no cell shrinkage or swelling as seen in respectively apoptotic or necroptotic cells [121, 122]. Genetic deletion of *Gpx4* (glutathione peroxidase 4), the main enzyme protecting cells from ferroptosis [120, 123], in KCs induces dysmorphic HFs, focal alopecia and dermatitis in mice. However, these phenotypes are transient in nature and mice lacking GPX4 in KCs have a normal lifespan [124]. So far, there is no in vivo proof directly linking ferroptotic cell death to inflammatory skin conditions. However, downregulation of GPX4 has been demonstrated in lesional skin of psoriatic patients, and treatment with the ferroptosis inhibitor ferrostatin-1 was shown to alleviate psoriasiform inflammation in mice [48]. Whether ferroptotic cell death is a cause or consequence of inflammation in these psoriatic plaques remains to be investigated.

### Autophagy

Macro-autophagy (hereafter referred to as autophagy) is the major cellular machinery for the recycling of amino acids. While this programme serves as an important pro-survival mechanism, autophagy can also lead to cell death. Constitutive ablation of genes essential for autophagy results in embryonically lethal phenotypes, indicating the crucial role of this process in tissue development (reviewed in [125]). Impairment of key autophagy genes in KCs demonstrated a crucial role for this process in the terminal differentiation programme that is initiated in the EpdSC niche. Indeed, KC-selective ATG (autophagy-related gene) 7 or ATG16L1-deficient mice exhibit enhanced sensitivity to DNA damage, altered lipid composition and a thickened cornified layer, pointing to defects in the desquamation programme [25, 126, 127] (Table 1). A defective autophagic flux resulting from depletion of ATG16L1 in KCs, leads to precocious anagen induction due to enhanced cell death rates, indicating that the apoptotic death of KCs impacts on the activation kinetics of HFSCs [25]. Also ATG9A has recently been identified as a critical autophagy mediator for maintaining skin homeostasis. Removal of this factor from KCs results in substantial epidermal cell death and spontaneous dermatitis due to improper TNF detoxification, identifying a novel control mechanism preserving skin integrity [128].

Polymorphisms in human *ATG16L1* have been linked to the dermatological conditions psoriasis vulgaris and palmoplantar pustulosis, further suggesting a role for keratinocyte autophagy in skin immunity [129, 130]. GWAS analyses of alopecia areata patients demonstrated associations with genes involved in autophagy, such as *STX17* (Syntaxin 17) [95]. Together, these observations demonstrate that autophagy plays important roles in KCs, protecting them from TNF toxicity and safeguarding the molecular clock that regulates HFSC proliferation.

## The impact of cell death on skin regeneration

**Fig. 4:**
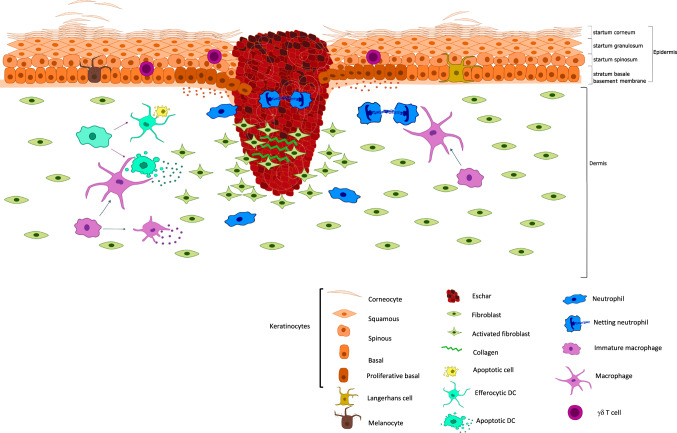
Role of different cell death modalities in cutaneous injury repair. Cell death is an inherent part of regenerative skin responses. After clot formation an inflammatory response mediates the infiltration of immune cells into the skin. Crosstalk between KCs and immune cells is crucial in recruiting a range of innate immune cells to the wound site. These cells signal to KCs to proliferate and migrate into the granulation tissue. When re-epithelialisation is finalized, skin-infiltrated immune cells including macrophages and dendritic cells play a dual role in the resolution of inflammation due to their phagocytotic capacities and production of anti-inflammatory cytokines. Alongside cell death by apoptosis, other forms of regulated cell death, such as NETosis, impact wound healing dynamics of the skin.

Epithelial tissue repair depends on the activation of tissue-resident SCs [131]. Cell death responses are crucial in orchestrating the highly complex process of regeneration, eliminating immune cells recruited to the wound site and counterbalancing the accelerated epithelial proliferative state. Recent findings shed light on how cell death shapes the activation of SCs during regeneration. When skin is wounded, the epidermal barrier is breached and cytokines, chemokines and danger-associated molecular patterns (DAMPs) are released, inducing an immune response that assists in injury repair [5]. The first immune cells that infiltrate wounded skin in large numbers are neutrophils that are recruited by KCs secreting HMGB1 (high mobility group box-1), a prototypical DAMP [132]. These neutrophils can execute a specific cell death pathway involving the production of neutrophil extracellular traps (NETs), a process that is highly prominent in diabetic wounds. NETs serve as a bacterial defence mechanism, but are detrimental to cutaneous injury repair [47, 133]. Genetic and pharmacologic targeting of HMGB1 in mouse KCs suppresses NET formation and accelerates wound closure in normoglycemic and diabetic skin [132, 134]. Besides neutrophils, macrophages and dendritic cells infiltrate injured skin and contribute to healing not only by promoting inflammation, but also by acting as phagocytes removing dead cells through efferocytosis. These macrophages die by apoptosis during the skin repair process, hence, preventing apoptotic responses in this cell lineage significantly delays injury repair [135] (Fig. 4).

Activities of the effector caspases-3 and -7 are crucial for skin regeneration due to their capacities to eliminate damaged cells and release proliferative signals [136]. However, mice deficient for the pro-apoptotic protein ARTS exhibit accelerated cutaneous wound repair [137]. While apoptosis is paramount for skin regeneration, it is clear that proper control of apoptotic cell death rates determines the healing kinetics. This is also exemplified in a mouse model in which the crucial cell death regulator cFLIP can no longer be cleaved by caspase-8, resulting in exaggerated cell death and a significant delay in skin regeneration [138]. Not only do extrinsic apoptotic pathways mediate skin regeneration, but the deregulation of intrinsic apoptotic signalling in HFSCs can accelerate cutaneous wound responses [139]. Future research on other regulated cell death modalities and the mechanisms that keep them in check during tissue regeneration holds promise for novel therapeutic approaches in management of wound care.

Proper efferocytosis by macrophages and dendritic cells present in wounded skin is crucial for injury repair due to its anti-inflammatory effects [33, 140] (Fig. 4). Inhibition of efferocytosis receptors, such as AXL and TIMD4, delay the healing kinetics of murine wounds and upregulation of pathways mediating cell clearance has been shown in cutaneous mouse wounds and human diabetic wounds [141]. When apoptotic cell death rates surpass the phagocytic capacity of cells present in the microenvironment, secondary necrosis resulting in lytic cell death can occur [142]. While the mechanisms mediating efferocytosis have mainly been described in professional phagocytes, a role for KCs and fibroblasts as occasional phagocytes in skin is emerging. Indeed, phagocytosis of dying/dead cells by basal KCs of the HF has been described during the catagen stage of hair cycling and recent findings show that efferocytosis by HFSCs impacts their regenerative capacities [23, 143].

## Cell death and skin tumour formation

While cytotoxic cancer therapies aim to kill tumour cells, cell death responses can induce proliferation of neighbouring stem cells [144] and even stimulate the repopulation of tumours undergoing radiotherapy [145]. In melanoma, cancer cells that escape apoptosis gain migratory and metastatic capacities [146]. So, while cell death is a crucial tumour-suppressive mechanism by removing (pre)malignant cells, it can also promote tumorigenesis by stimulating inflammatory, regenerative and even metastatic responses. Skin cancers also adhere to this duality. Overexpression of the anti-apoptotic protein BCL-XL sensitizes mouse KCs to carcinogenesis and malignancy [147]. Genetic alterations in KC survival pathways, for example those resulting in constitutive activation of AKT, accelerate skin tumour formation [148]. In carcinogenic conditions, TNF-mediated signalling pathways are crucial in determining the propensity of KCs to either proliferate or die. Mice deficient for both TNF receptors are resistant to chemically-induced skin cancer protocols [149]. Along these lines, NF-κB blockade by overexpression of the NFκB-inhibitor IκBα in human KCs promotes their transformation to squamous cell carcinomas (SCCs) and in mice, these conditions result in increased KC apoptosis and spontaneous SCC formation [150, 151]. A recent genetic screen identified several hits in the TNF signalling cascade sensitizing melanoma cells to elimination by T-cells, demonstrating the potential of manipulating cell death pathways in immune checkpoint blockade [152].

As is the case for apoptosis, necroptosis and pyroptosis can have either tumour-promoting or -suppressing effects in the skin, depending on the tumour type and stage. In various tumour types including malignant melanoma, necroptotic mediators are strongly suppressed, indicating acquired necroptotic resistance of tumour cells [153]. In line with this, senescent melanocytes that are capable of resisting cell death, are emerging as a potential cell-of-origin in melanoma and specific targeting of factors released by senescent melanocytes are showing promise in counteracting melanoma [154, 155]. In keratinocyte-derived tumours, such as head and neck SCC, a substantial amount of necroptosis has been reported and high levels of phosphorylated MLKL were shown to correlate with lymph node metastasis and tumour progression [156]. Intriguingly, naked mole-rats- a species known for its remarkable resistance to skin carcinogenesis- lack the necroptotic mediator RIPK3, suggesting a putative role for necroptosis in neoplastic skin responses [157]. This study also demonstrates that pharmacological or genetic inhibition of necroptosis delays chemically-induced skin carcinogenesis in mice [157]. However, more research is needed to elucidate whether, when and how specific cell death programmes should be targeted to enable more efficient and safer removal of tumour cells.

## Conclusions

Collectively, the tight balance between keratinocyte proliferation and removal is crucial for the skin’s barrier function, but also for proper cycling of HFs. While periodic bouts of cell death are inherent to the HFSC niche, the abundant presence of dying cells in the EpdSC niche is detrimental to proper epidermal barrier function. While there is no doubt that apoptosis represents a crucial cell death modality important for skin homeostasis, hair cycling, and for counterbalancing tumour formation, a growing body of evidence points to important contributions of other regulated cell death programmes in these processes. The consequences of distinct cell death programmes on the survivor cells within specific SC niches are beginning to unravel as our understanding of the molecular executioners of these programmes increases. This allows us to pinpoint more precisely which types of cell death occur in the different SC compartments of the skin. However, our understanding of why certain progenitor cells are more sensitive to specific types of cell death has only recently begun to unfold. It remains unclear how this sensitivity is regulated and potentially altered in disease. Understanding these complex concepts holds the key to revealing the therapeutic potential of manipulating cell death programmes within the appropriate tissue context, thereby advancing clinical treatments of skin diseases.
