# Publisher Correction: Cell death as an architect of adult skin stem cell niches

**DOI:** 10.1038/s41418-024-01362-x

**Published:** 2024-09-02

**Authors:** Kim Lecomte, Annagiada Toniolo, Esther Hoste

**Affiliations:** 1 VIB Center for Inflammation Research, 9052 Ghent, Belgium; 2 Department of Biomedical Molecular Biology, Ghent University, 9052 Ghent, Belgium

**Keywords:** Cell biology, Cell death and immune response

Correction to: *Cell Death & Differentiation* 10.1038/s41418-024-01297-3, published online 22 April 2024

In this article Kim Lecomte and Annagiada Toniolo were indicated as co-first authors. This information has been omitted during the production process. The original article has been corrected.

